# Functional HLA-C expressing trophoblast spheroids as a model to study placental–maternal immune interactions during human implantation

**DOI:** 10.1038/s41598-022-12870-6

**Published:** 2022-06-17

**Authors:** Marina Alexandrova, Diana Manchorova, Yuan You, Gil Mor, Violeta Dimitrova, Tanya Dimova

**Affiliations:** 1 Institute of Biology and Immunology of Reproduction “Acad. K. Bratanov”, Bulgarian Academy of Sciences, Sofia, Bulgaria; 2 C.S. Mott Center for Human Growth and Development, Wayne State University, Detroit, USA; 3 Medical University, University Obstetrics and Gynecology Hospital “Maichin Dom”, Sofia, Bulgaria

**Keywords:** Tissue engineering, Cell death and immune response

## Abstract

In healthy couples over half of the conceptions result in failed pregnancy and around 30% of them occur during implantation defining it as a rate-limiting step for the success of native and in vitro fertilization. The understanding of the factors regulating each step of implantation and immune recognition is critical for the pregnancy outcome. Creation of 3D-cell culture models, such as spheroids and organoids, is in the focus of placental tissue engineering in attempt to resemble the in vivo complexity of the maternal-fetal interface and to overcome the need of laboratory animals and human embryos. We constructed stable, reliable, and reproducible trophoblast Sw71 spheroids which are functional independently of the serum level in the culture media. These models resemble the hatched human blastocyst in size, shape and function and are useful for in vitro studies of the in vivo concealed human implantation. Since Sw71 spheroids produce HLA-C, the only classical MHC molecule indispensable for establishment of the immune tolerance and proper human implantation, they are applicable for the evaluation not only of implantation itself but also of maternal-trophoblasts immune interactions. In addition, Sw71-blastocyst-like spheroids are manipulable in low-volume platform, easy to monitor and analyze automatically under treatment with favorable/detrimental factors.

## Introduction

Human implantation is a highly complex and still unsolved phenomenon by which the conceptus is transported to its site in the uterus, oriented properly, and then attached by adhesion and trophoblastic penetration into the receptive and modified endometrium (decidua) about day 6 post-conception^1^. In healthy couples over half of the conceptions result in failed pregnancy and around 30% of them occur during implantation defining it as a rate-limiting step for the success of native and in vitro fertilization (IVF)^2–4^. Aberrant implantation can cause a variety of clinical disorders including miscarriage, intrauterine growth retardation and pre-eclampsia^5^⁠. Except biological, human implantation is an immune paradox because the semi-allogeneic embryo is normally accepted, indicating the presence of active mechanisms of immune tolerance and/or fetus strategies to avoid an attack by the maternal immune system. Such a strategy is the lack of HLA class I molecules on the villous trophoblasts and their atypical expression by the extravillous trophoblasts (EVTs) contacting with maternal immune cells into decidua. In uncomplicated pregnancies EVTs invade through the decidua and express the non-classical (invariant) HLA class I molecules HLA-G and HLA-E^6,7^⁠ and the classical polymorphic HLA-C molecule^8,9^⁠. The paternal inherited HLA-C variants, expressed on the invading trophoblast are recognized by Killer Ig-like Receptors (KIRs) on maternal immune cells such as cytotoxic T and NK cells⁠^8,9^. Some maternal/fetal KIR/HLA-C combinations might be favorable to trophoblast cell invasion and protective for implantation, and others could initiate allo-responses or insufficient placental growth^8,9^⁠. Within the context of the current state of knowledge, human implantation remains an obscure process and real barrier to assisted reproduction techniques, introduced many years ago^10^⁠ and therefore, the possibility for clinical intervention is still limited. Thus, understanding the factors regulating each step of implantation and immune recognition is critical for improving pregnancy outcome. The mode of implantation in humans differs considerably from other mammalian species resulting in the restricted range of functional experimental models^8,11^⁠. Regulations around the world including USA and EU countries like Bulgaria, restrain in vitro studies using human blastocysts^12,13^⁠. Ethical constraints and restricted access to early gestational tissue strongly limit our knowledge of the human placenta in the earliest stages of implantation. Thus, human trophoblast models, that adequately mimic trophectoderm development during implantation, are urgently needed for “better understand pregnancy loss and infertility without experimenting on human embryos”^14^. Traditional two-dimensional (2D) cell culture models lack physiologically relevant environmental conditions in terms of physical and biochemical settings. Therefore, studies turned to construction of three-dimensional (3D) organoid and spheroid systems where cells benefit from cell to cell and cell to extracellular matrix (ECM) contacts and exist in a more biochemically relevant state with gradients of oxygen, nutrients, and metabolites^15,16^. Different types of cells were used including cancer cell lines, embryonic stem and reprogrammed to stem somatic cells. They all present advantages and disadvantages and show variating success for formation of blastocyst-like structures^17–19^. In a series of elegant experiments, G. Mor’s group created 3D trophoblast cell line Swan71 (Sw71) and blastocyst-like structures during the peri-implantation period^20–22^. In this study we extended the data on the morphology and behavior/function of the 3D Sw71 models in microenvironment with different serum level and tested their HLA-C expression/production in view of the applicability of this model for the evaluation of maternal–trophoblast immune interactions in (un)successful human implantation.

## Results

### Monitoring of 3D Sw71 trophoblast models’ generation and morphology

#### Generation of 3D spheroids

**Figure 1:**
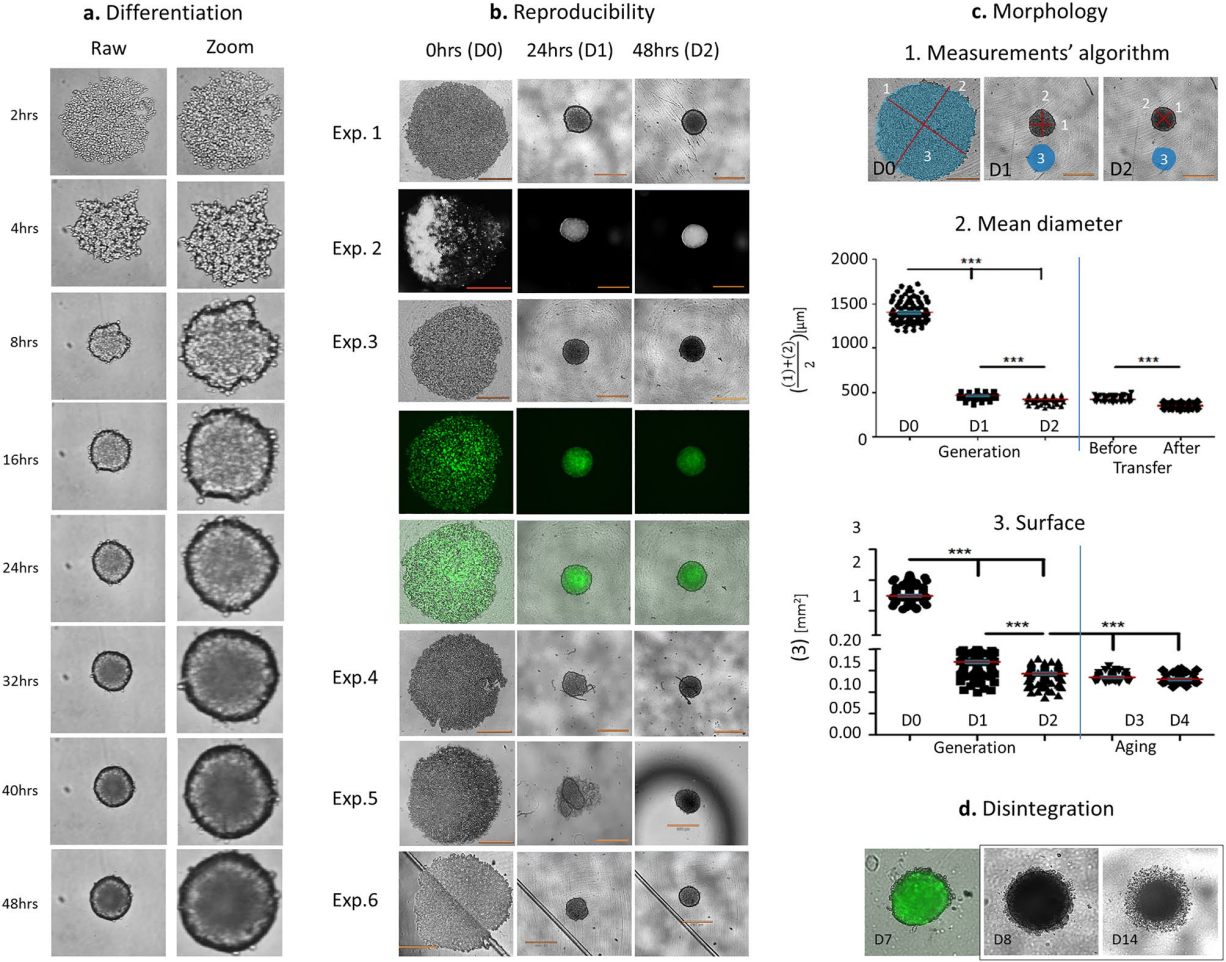
Generation of Sw71 spheroids as a model of early human placenta. (**a**) Differentiation of spheroid from Sw71 cells visualized at different time-points: 2–8 h—aggregation; 8–24 h—compaction, 24–48 h—stable spheroid. Left column—formation of a single differentiated spheroid with intact border, right column—additional digital zoom to show the formation of intact periphery. (**b**) Reproducibility (exp. 1–3) and some obstacles (exp. 4–6) during differentiation of the models exemplified by representative Sw71 spheroids captured at D0, D1 and D2 (6 independent experiments): exp. 1—fine spheroid, brightfield view; exp. 2—fine spheroid, phase contrast view; exp. 3—fine Sw71-GFP spheroid, trans, fluorescent and overlaid view; exp. 4—mechanical non-organic contamination; exp. 5—nonoptimal humidity; exp. 6—plate defects. (**c**) Sw71 spheroids’ morphological characteristics: 1. Measurements; 2. Mean diameter values of Sw71 spheroids (n = 126) at D0, D1 and D2, and before and after their transfer; 3. Surface measured for the same spheroids. (**d**) Sw71 spheroids’ disintegration during long term culture without additional feeding presented by two representative spheroids. Left—Sw71-GFP spheroid on D7 in overlayed view. Right—Sw71 spheroid, pictured on D8 and D14. Imaging: (**a**) time-lapse live cells imaging system OMNI (CytoSmart Technology, The Netherlands), magnification 10× (+ manual digital zoom), pictures taken every 2 h, but only selected images were included. (**b**, **c**, **d**) ECHO Revolve microscope (RVL-100-M, Echo, San Diego, CA, USA), magnification 4×. Bars (**b**, **c**) = 530 µm. Statistics was processed with GraphPad Prism v.5.0 software.

The reproducibility of the establishment of 3D Sw71 spheroids was confirmed by 30 independent experiments performed mainly by two operators. We obtained over 70% viable spheroids from each experiment. Within 48 h we obtained a stable, self-assembled, round shaped, multi-layer relatively symmetric 3D spheroids (roundness index = 0.96 ± 0.04). The Sw71 cells cultured in a U-bottom 96 well low attachment plates initially formed loose aggregate (0–8 h), followed by compaction (12–24 h) and formation of single differentiated, stable spheroid with an intact periphery (24–48 h, Fig. 1a). The spheroids were monitored with or without a live cell imaging system; in bright, phase contrast, fluorescent modes, overlayed visualizations were easily obtained (experiments 1–3, Fig. 1b). Interestingly, the presence of non-organic contamination (dust, filaments etc.) in the wells did interfere with the shape and size but did not disturb the aggregation and formation of the 3D structures (experiment 4, Fig. 1b) as well as their functionality. We found that the spheroids’ generation process was particularly sensitive to humidity. In absence of optimal moisture, spheroids disintegrated into cell clusters, and when the humidity was normalized, their structure was restored (experiment 5, Fig. 1b) with preserved functionality in 97% of such rescued spheroids migrated. In general, plate defects did not interfere with spheroids’ differentiation, but compromised the quality of the images (experiment 6, Fig. 1b).

#### Morphology of Sw71 spheroids

Our next objective was to characterize the spheroids by determining three morphological parameters—mean spheroid diameter, surface, and volume. Measurements from 10 independent experiments including over 100 spheroids was performed and exemplified in Fig. 1c.1. We determined the average of the two largest perpendicular diameters, the area using the ECHO software and the formula V = 0.52 × 22 × 1 (Fig. 1c.1). Results revealed that on day 0 (D0) the mean diameter of the spheroids was 1.402 ± 110.60 mm, and the average surface was 1.487 ± 0.234 mm^2^. Between D0 and day 2 (D2) significant compactization was noted as the spheroids decreased in size to 466 ± 28.45 µm diameter and 0.170 ± 0.023 mm^2^ surface on day 1 (D1) and 425 ± 27.14 µm diameter and 0.143 ± 0.018 mm^2^ surface at D2 (Fig. 1c.2,c.3 respectively, *p* = 0.0001). The mean spheroid volume showed the same tendency (1.492 ± 0.355 mm^3^ at D0, 0.055 ± 0.009 mm^3^ on D1 and 0.041 ± 0.007 mm^3^ at D2, *p* = 0.0001). Once achieving compactization on D2, the fully differentiated spheroids can be transferred from the ULA plate to other plates, maintaining their integrity in spite of the manipulations. Interestingly, right after transfer to fresh media we can observe a significant decrease in size to 348 ± 30.99 µm diameter (Fig. 1c.2). When the models were kept in the ULA plate for a longer period (until day 4) without additional feeding, a considerable shrink was observed (Fig. 1c.3). The spheroids were very stable, maintaining their morphology up to 7–8 days when first signs of spheroid disintegration were observed. Around day 14 a large halo with de-attached dead cells in the periphery of the spheroids was present (Fig. 1d). Consequently, we demonstrate that the spheroids are functional and available for experiments 2 days after their differentiation and remain viable with preserved morphology up to 2 weeks.

### Assessment of the function of Sw71 trophoblast spheroids

#### Attachment on epithelial endometrial cells

**Figure 2:**
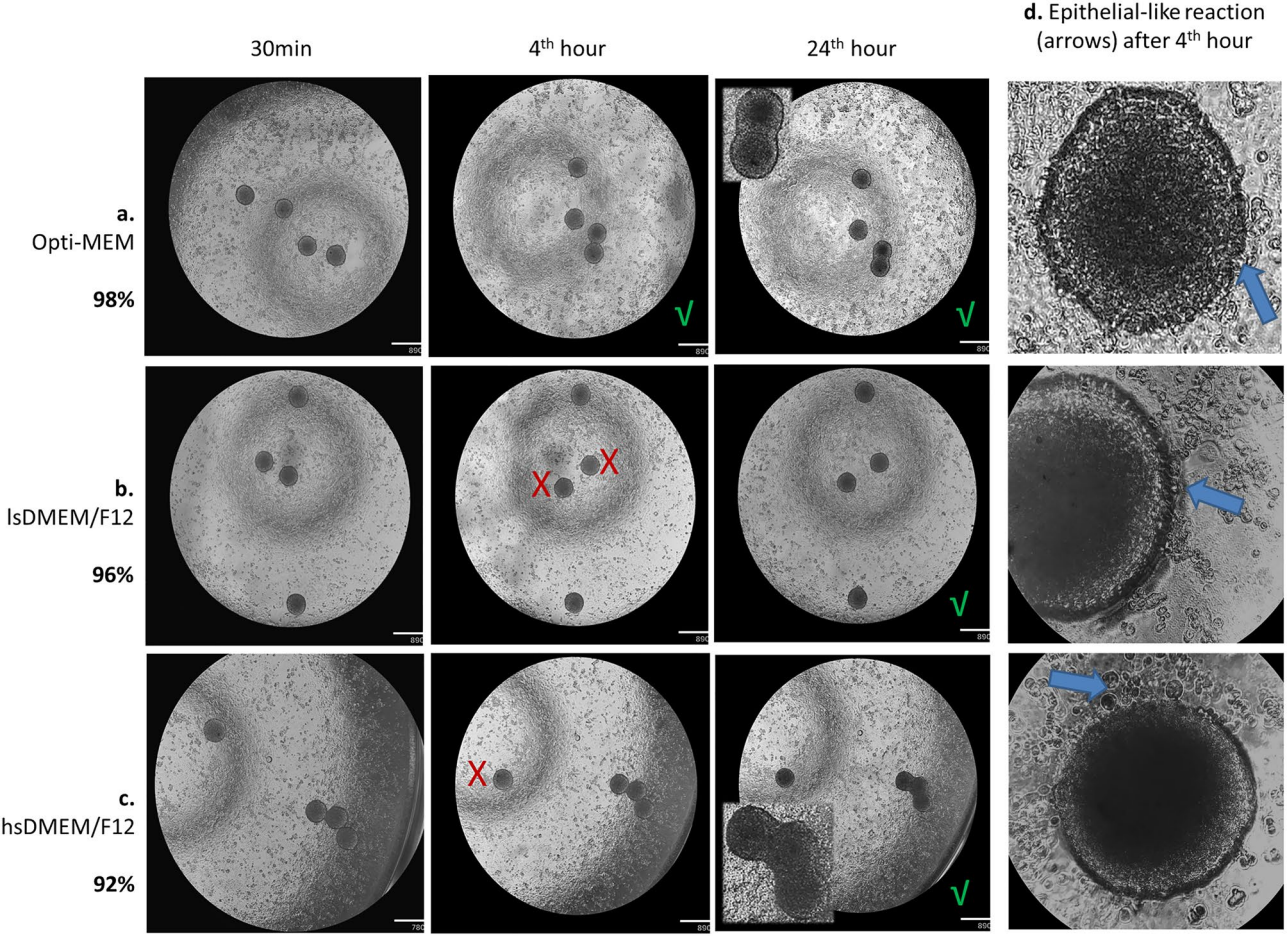
Sw71 spheroids’ attachment capacity. (**a**–**c**) Representative Sw71 spheroids showing 100% attachment rate 24 h after co-culture with HEC-1A epithelial cells with best results in Opti-MEM followed by lsDMEM/F12 and hsDMEM/F12 (98%, 96% and 92% respectively). Note that closely disposed spheroids merged as co-incubation advanced (inserts in (**a**) and (**c**), 3rd column). Presented images were taken after shake at 30th min, 4th and 24th hour of co-incubation. (**d**) Representative spheroids in corresponding medium, with focus on the border with signs of epithelial-like reaction (arrows) after the 4 h of co-culture. Imaging: Echo Revolve microscope (Echo, San Diego, USA), magnification 4× (**a**–**c**), 4x + digital zoom (**d**, row 1) and 20× (**d**, row 2 and 3). HEC-1A cells confluency was over 95%, estimated by automated live cells imaging system OMNI (CytoSmart technology, The Netherlands). X—unattached spheroid; √—all spheroids were attached at the current time point, bars (**a**–**c**)—890 µm. Seven independent experiments were performed.

The first step in blastocyst implantation is the adhesive interaction between the trophoblast and endometrial epithelium. This is a crucial step for the blastocyst penetration and subsequent invasion/migration during successful implantation. To evaluate the capacity of the spheroids to attach to the epithelium, we transferred differentiated spheroids on an epithelial cells monolayer and incubated the cultures for 24 h with horizontal shaking of 1000 or 3000 rpm at 1st, 2nd, 4th and 24th hour. Over 95% of the spheroids were attached to the epithelium proved by shake at 1000 rpm. We tested the impact of the media and observed that the highest number of the attached spheroids (98%) were those cultured in Opti-MEM condition compared to lsDMEM/F12 (96%) and hsDMEM/F12 (92%, Fig. 2a–c). Interestingly, when the shaking was increase to 3000 rpm we found an attachment rate as follows: 100% in Opti-MEM, 96% in lsDMEM/F12 and 88% in hsDMEM/F12. The earliest time-point for firm attachment of the spheroids to the epithelium regardless of the shake intensity and media’s serum levels was 4th hour (Fig. 2a–c) when we were able to observe an epithelial-like reaction at the site of the spheroid attachment (Fig. 2d).

#### Migration and invasion capacity

**Figure 3:**
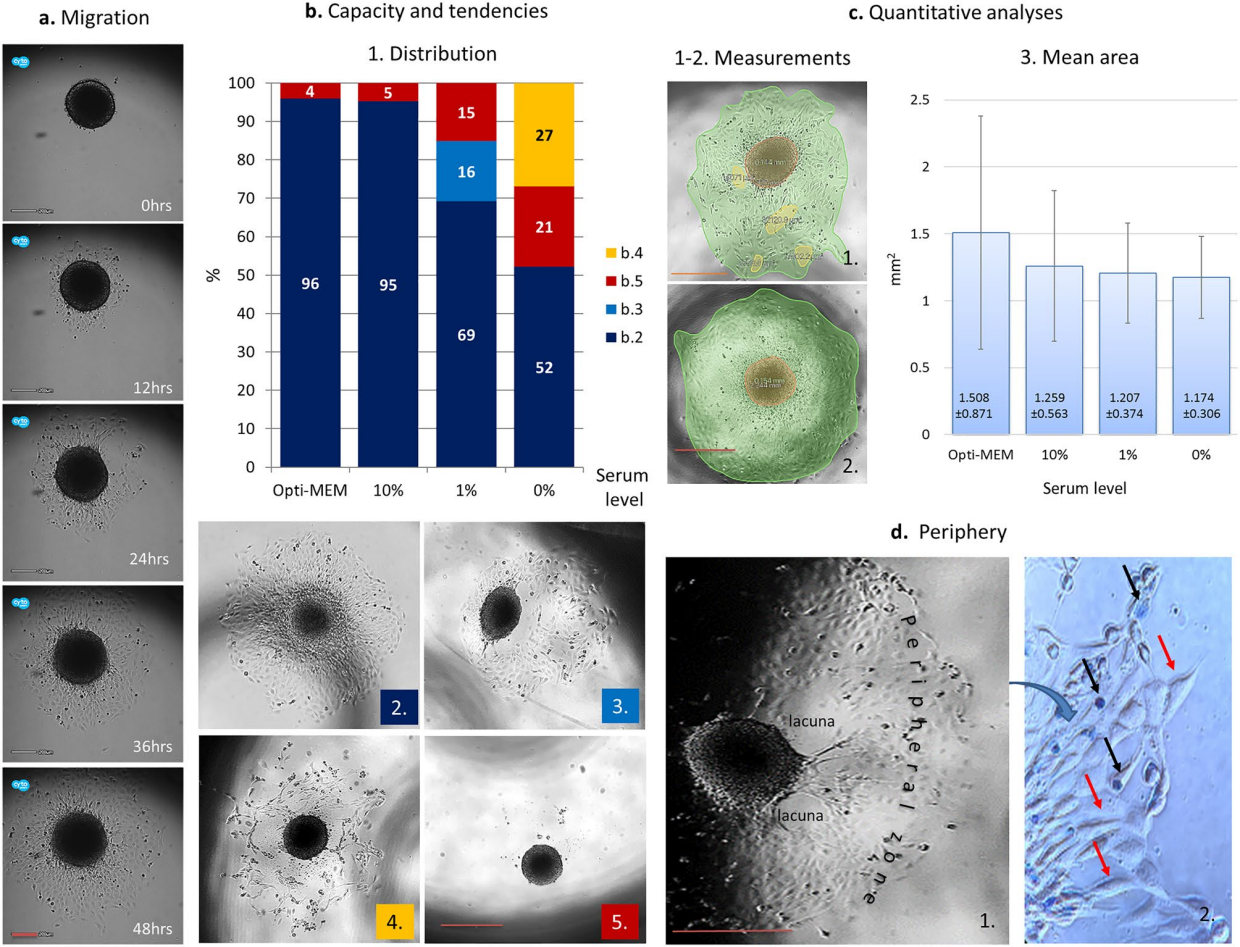
Sw71 spheroids’ propensity for migration. (**a**) Time-lapse imaging of migrating spheroid (48 h). (**b**) Analyses of the migration capacity of Sw71 spheroids placed in microenvironment with differing serum levels: 1. Percentage of the migrating spheroids. 2–5. Corresponding representative images: 2—radial migration; 3—fine radial migration with interruption on D2; 4 and 5—non-migrating spheroids with peeling of dead peripheral cells; (**c**) Migration area—quantitative analysis: 1–2: Examples for measurement of the migration area (green) that equals the area without the remaining spheroid surface (red) and the present lacunae areas (yellow) if any. 3. Migration area (mean ± SD) in each of the tested media. (**d**) Morphology of the periphery of migrating spheroid: 1. Detailed view of migrating spheroid at 60th hour with defined lacunae and peripheral zone; 2. Closer view of the peripheral migration zone, stained with Trypan blue, showing migrating alive cells, elongated in shape and colorless (red arrows) and dead cells, round and blue (black arrows). Imaging: (**a**) time-lapse live cells imaging system Lux (CytoSmart Technology, The Netherlands), magnification 10× plus digital zoom. The images were taken every 2 h, but only selected images were included. (**b**–**d**) Echo Revolve microscope (Echo, San Diego, USA), magnification 4× plus digital zoom (**b**, **c**, **d**-left) and 20× (**d**)-right. Bars: (**a**) 200 µm; (**b**–**d**) 530 µm. Twenty independent experiments were performed.

Trophoblast migration and invasion into decidua is a major step on establishing placentation. These important events determine the ability of trophoblasts to reach the glandular histotrophic nutrition and then by invading deeply to remodel uterine spiral arteries and thus to establish functional placenta. To determine the migration properties of Sw71 spheroids we transfer single spheroids to regular tissue culture 96 well plated and monitored them for 48 h using life imaging. Time lapse analyses revealed that the spheroids attached within 10–12 h, followed by a well synchronized migratory process, where trophoblast cells emerged from the spheroid’s periphery and continued migrating throughout the surface of the well (Fig. 3a). The highest migration capacity of Sw71 spheroids was detected in Opti-MEM (96%), followed by hsDMEM/F12 (95%) condition, but the serum reduction from 10 to 1% decreased the migration rate to 85% (in lsDMEM/F12). Moreover, 16% of these migrations were characterized with formation of large central lacuna (Fig. 3b.3). In the serum-free media (sfDMEM/F12) 52% of the tested spheroids migrated (Fig. 3b.1), 27% showed initial migration at D1 interrupted on D2 (Fig. 3b.4) and 21% did not migrated at all. In general, 15% of all tested spheroids did not migrate at all, most of them being in no or low serum conditions (Fig. 3b.5). The most frequent migration pattern was radial (69%), independent of the media or serum concentrations (Fig. 3b.2). The quantitative analysis showed no significant difference in the spheroids’ migration area on D2, regardless serum content (*p* = 0.59, Fig. 3c). When stained with Trypan blue for vitality most of the cells migrating out of the spheroid were alive, elongated in shape and colorless, but some dead cells were also present, being round and blue (Fig. 3d).

**Figure 4:**
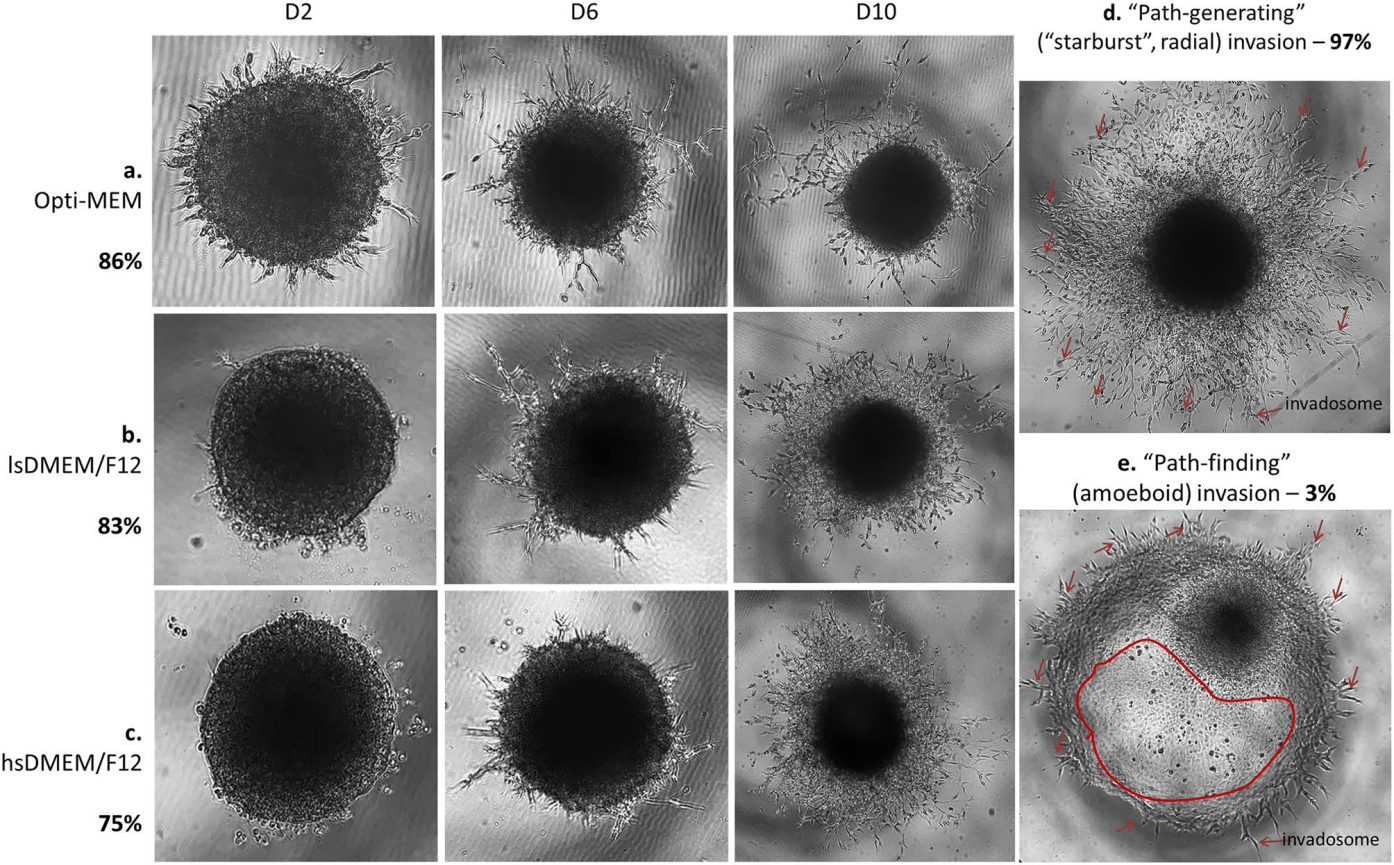
Sw71 spheroids’ propensity for invasion. (**a**–**c**) Representative triplets of invading Sw71 spheroids. Optimal results were gained in Opti-MEM followed by lsDMEM/F12 and hsDMEM/F12 (86%, 83% and 75% respectively). (**d**) Representative spheroid on D10 in hsDMEM/F12, showing a classical “path-generating” radial invasion pattern, seen in 97% of the cases. (**e**) Representative spheroid on D6 in hsDMEM/F12, showing a non-typical “path-finding” amoeboid invasion (zone marked in red: cells with rounded, deformable morphology squeeze through narrow spaces and smaller pores of the Matrigel). Of note, invading Sw71 spheroid trophoblasts form invadosomes (orange arrows) for the proteolysis-dependent ECM degradation which are numerous and well-developed in radial invasion (**d**) and only a few in the amoeboid invasion (**e**). Imaging: Echo Revolve microscope, Echo, San Diego, CA, USA, magnification 4× ± digital zoom. Seven independent experiments were performed.

To test the capacity of the spheroids to invade, we transfer single spheroids to the top of 50% Matrigel in different media conditions and monitored them using life imaging. Over 81% of Sw71 spheroids showed propensity for spontaneous invasion independent of the medium. The highest invasion rate was found in Opti-MEM culture (86%) compared to lsDMEM/F12 (83%) and hsDMEM/F12 (75%) cultures (Fig. 4a,b,c). The invasion emerged after D2 and was characterized by flattening out of the trophoblast cells from the periphery of the spheroids and formation of invadosomes (Fig. 4d,e). These invasive projections penetrate the Matrigel and continue growing in radial (“path-generating”) mode in a time-dependent manner. Although we detected amoeboid (“path-finding”) type of invasion in some spheroids (2 out of 75 tested, 1 in hsDMEM/F12 and 1in lsDMEM/F12, Fig. 4e), the radial pattern of invasion was the most observed (Fig. 4a–d). The invasion process could be observed up to 10 days, especially in hsDMEM/F12 cultures. In summary, Sw71 spheroids demonstrate pronounced invasiveness and ability to form projections that resemble chorionic villi structures (Fig. 4c).

#### Sw71 spheroids express and produce HLA-C molecule

**Figure 5:**
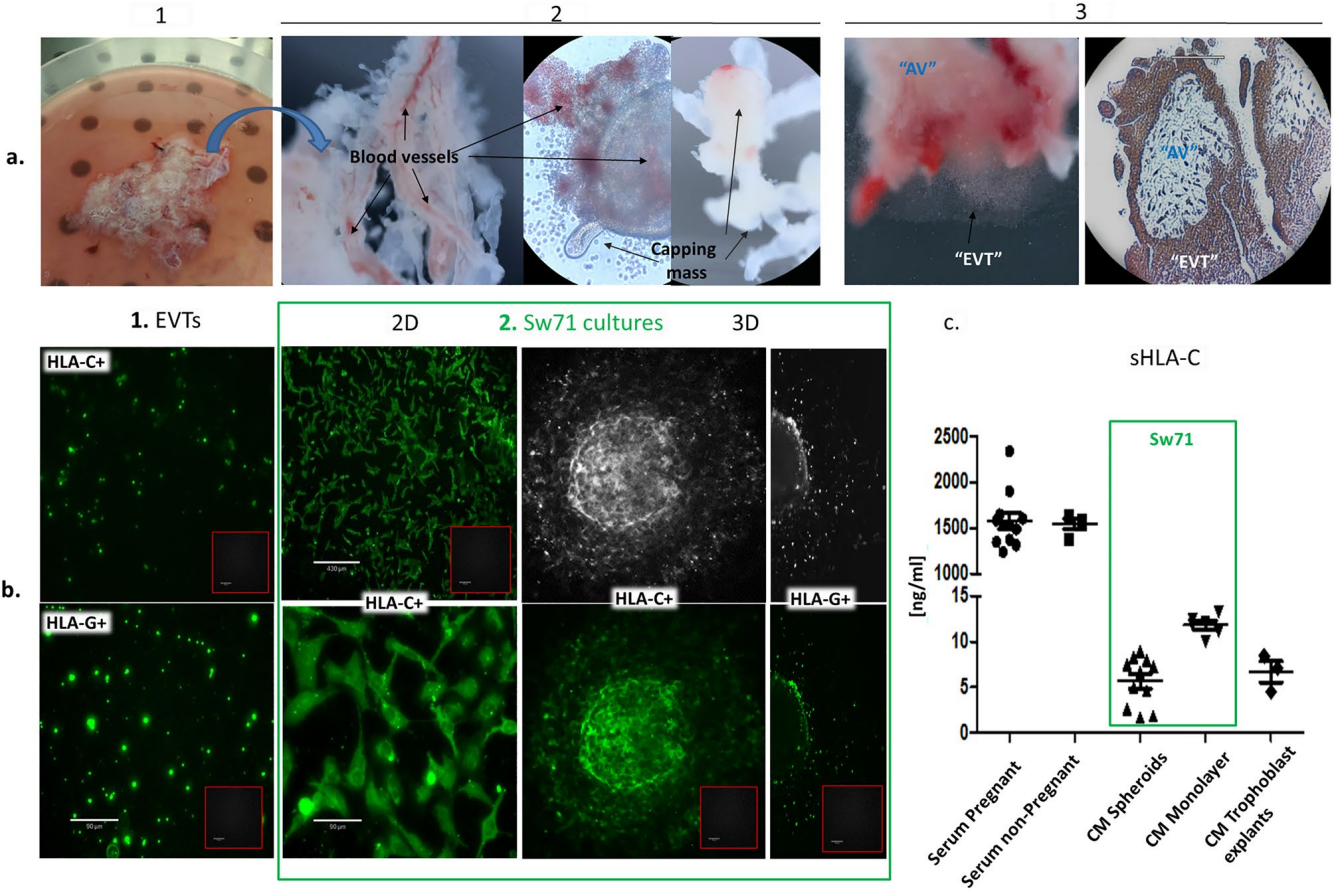
HLA-C expression/production by Sw71 cells, Sw71 spheroids and primary trophoblasts. (**a**) Early placenta-derived trophoblast explants as primary “ex vivo” model of implantation: 1. Trophoblast in a petri dish to be cut into small explants; 2. Trophoblast explants cultured in hsDMEM/F12 with signs for vitality—blood vessels, formation of capping mass, expansion of EVTs; 3. Vital, attached trophoblast explants with expanding EVTs (left) resembling anchoring chorionic villa with trophoblast column on the tips, containing EVTs (right, paraffin slide). The EVTs within the trophoblast column are HLA-C-positive (brown). (**b**) Immunocytochemical staining for HLA-C and HLA-G: 1. Explants-derived EVTs expressed HLA-C (up) and HLA-G (down); 2. Sw71 cultures: HLA-C + Sw71 cells in monolayer (2D) and migrating Sw71-GFP spheroids positive for both markers HLA-C and HLA-G (3D, left and right columns, respectively). The spheroids were captured in brightfield and fluorescence mode. Inserts in (**b**)—negative controls for the immunofluorescent staining where the primary antibody was omitted. (**c**) sHLA-C in CM and sera, measured by ELISA. Imaging: Echo Revolve microscope (Echo, San Diego, CA, USA), magnification 4× (**a**) and 20× (**b**).

Trophoblast cells are the primary fetal cells directly in contact with the maternal immune system and expression of HLA-C is a critical component of proper immune recognition. Our next step was to evaluate whether Sw71 spheroids express/produce HLA-C molecule as compared to early placenta chorionic villi (Fig. 5a,b). First, we cultured early placenta-derived explants for 72 h to obtain EVTs and confirm their HLA-C expression. The human trophoblast explants remained vital for several days as proven by the presence of well visible blood vessels and the formation of capping mass (Fig. 5a.2). Following attachment, placental explants formed structures resembling the anchoring chorionic villi around which the expansion of EVTs was observed. As shown these EVTs were positive for trophoblasts markers HLA-C (Fig. 5a.3, left and Fig. 5b.1, up) and HLA-G (Fig. 5b.1, down). In addition, we stained paraffin embedded trophoblasts tissue for HLA-C. As shown in Fig. 5a.3, an anchoring villa of human early placenta containing trophoblasts column with EVTs positive for HLA-C. Then we stained Sw71 cells in monolayer as well as Sw71 spheroids for HLA-C. The results showed strong HLA-C reaction in both Sw71 cells as monolayer (2D, Fig. 5b.2) and in Sw71 spheroids (3D, Fig. 5b.3), including in the cells migrating from the spheroids. We stained in the same manner for HLA-G and as found Sw71 spheroids expressed HLA-G (Fig. 5b, last column)**.** Next, we evaluated the capacity of the Sw71 2D and 3D cultures to produce soluble HLA-C (sHLA-C). We measured HLA-C in the supernatants from these cultures by ELISA. Both, monolayer, and spheroids express detectable levels of sHLA-C (11.80 ± 1.13 ng/ml, n = 6 and 5.68 ± 2.66 ng/ml, n = 11, respectively (Fig. 5c). Of note, Sw71 spheroids secreted comparable amount of sHLA-C to that of primary trophoblasts (5.68 ± 2.66 ng/ml vs. 6.7 ± 1.99 ng/ml, n = 3, *p* = 0.76, Fig. 5c) independently of the serum level in the medium of both cultures (1% for Sw71 spheroids and 10% for the explants). Also, there was no significant difference in the HLA-C production by migrating and non-migrating Sw71 spheroids (5.76 ± 2.69 ng/ml, n = 7 vs. 5.54 ± 3.00 ng/ml, *p* = 0.95). The sHLA-C was abundantly present in sera of pregnant and non-pregnant women without any difference (1576 ± 299 ng/ml, n = 12 vs. 1547 ± 114 ng/ml, n = 4, *p* = 0.67, Fig. 5c).

## Discussion

In this study we constructed stable, reliable, and reproducible trophoblast Sw71 spheroids which are functional regardless the serum level in the culture media. These models were manipulable in low-volume platform and easy to monitor and analyze automatically. Importantly, Sw71 spheroids expressed and produced HLA-C molecule similarly to the native 3D trophoblasts explants derived from early pregnancy human placenta. The creation of three-dimensional cell culture model systems, such as spheroids and organoids, is in the focus of placental tissue engineering. They better resemble the complexity of the in vivo microenvironment at maternal–fetal interface (MFI) during human implantation and overcome the need for experimental work with specimens, presenting strong ethical and practical constraints^12–14^⁠. The successful implantation requires efficient apposition, adhesion/attachment, invasion and migration into decidual stroma, glands and blood vessels until the embryo is steadily implanted within the uterine mucosa^1,8,23^⁠. Blastocyst models using trophoblast-derived choriocarcinoma cell lines have been used extensively but these cells are malignant and tumorigenic, differing from primary tissues in trophoblast-specific markers, as well as human leukocyte antigen (HLA) status^24–26^⁠. Recently embryonic stem cells or reprogrammed to stem somatic cells were used for formation of blastocyst-like structures with higher accuracy in the morphology^17–19^ (presence of different cell types at low numbers, of “blastocoel” cavity) but they are hardly achievable for technical and ethical reasons thus limiting their use as experimental models in many laboratories. On the other hand, primary trophoblasts, isolated from first-trimester placenta, can hardly be manipulated since they rapidly cease proliferation and fade away. Here we confirm the successful, accessible, feasible and reproducible generation of spheroids from immortalized, non-cancer, first trimester trophoblast cell line Sw71^20–22^ with standard cell culture equipment and techniques. The yield of fine, uniform and differentiated Sw71 spheroids, resembling the shape, size, and behavior of the hatched human blastocyst during the peri-implantation period, was over 70% per experiment. As compared to unexpanded blastocyst within the zona pellucida^27,28^, Sw71 spheroids are about two times larger in diameter. It is important to note that except specific morphology and size the established 3D models for evaluation of human implantation must have the ability to attach, migrate and invade^22^. Sw71 spheroids in our hands showed functional capacity as “destined to implant” structures determined rather by their trophoblast origin, and independent of the microenvironment serum levels. Low (1%) and reduced (below 10%) serum levels promoted attachment and invasion while high serum levels (10%) facilitated spheroid migration. Although the Opti-MEM condition brought higher migration rate, and there were nice results in low and no serum media (also despite the lower rate), the serum is not the determinative factor for the migration process. Another important finding was that 10% FCS in the culture media protects the spheroids against unfavorable environmental factors. These results are in line with previous observations showing that serum supplementation may improve embryo culture outcomes but is not essential for IVF early embryo culture and transfer^29–31^⁠. Considering that the concentration in serum proteins in general is much higher than of those produced by mammalian cells in vivo, and that lower protein concentrations result in low levels of contaminants, we suggest lsDMEM/F12 and the most favorable condition—commercial Opti-MEM as fine conditions to culture Sw71 blastocyst-like models. The attachment is an important step during peri-implantation since failure to interact would lead to infertility^4,8,32^⁠. The uterine and trophoblast epithelium make their first contact via their apical cell poles for a short time within the window of implantation^33^. Attachment models presenting co-culture of cancer derived human endometrial epithelial cell lines (receptive: RL95-2 > HEC-1A > KLE; non-receptive AN3-CA etc.) and choriocarcinoma cell line-derived spheroids (BeWo, JAR and other) have been introduced before^34,35^⁠. Our HEC-1A-Sw71 attachment model profits from the luminal epithelium adenocarcinoma origin of the HEC-1A, defining them as epithelial, polarized cells with apical pole, expressing estrogen, progesterone, and androgen receptors and from the Sw71 spheroids’ imprint, closer to the developing embryo’s trophoblast^22,33,36^⁠. In addition, this cell line presented average receptivity^34,35^⁠. New finding in our study was the mechanical vibrations of 1000 or 3000 rpm applied during the attachment assay. These shaking forces mimic the in vivo environment, where the blastocyst is in a continuous movement due to the beating of the epithelial cilia in the fallopian tube and within the uterus close to the site of implantation. The beating of the cilia of the oviductal epithelial cells is found to be around 5–20 Hz^37^⁠, which is close to 1000 rpm (~ 17 Hz) shaking force. Moreover, the three-dimensional vibration of 56 Hz for 5 s every 60 min has been shown to increase the implantation rate in IVF^37–39^, a fact that we noted with our models when we applied 3000 (50 Hz) shaking force. Joint culture of 4 spheroids was chosen since in IVF this approach is known to improve the implantation rate. We, like others^34^, observed morphological changes in trophoblast spheroids’ peripheral zone as signs of epithelial-like reaction at 4th hour of co-incubation. Based on the high attachment properties (> 90%) of our Sw71 spheroids to a HEC-1A monolayer, we suggest them as an efficient model for adhesion-competent trophoblast. Further analyzes as scanning electron microscopy should be performed to give more details for this interaction. Both trophoblasts and tumor cells possess invasive behavior, but trophoblast invasion is tightly regulated and ceases once reaching the spiral arteries within the uterine myometrium^40^. Pregnancy disease states like recurrent miscarriage, preeclampsia and intrauterine growth retardation are often related to suboptimal trophoblast invasion within the placental bed^26,41,42^. On the other hand, extensive invasion may result in life-threatening conditions where trophoblast cells may rupture the uterus^43^. The ECM, which fills the space between cells and form the basal membranes through a complex organization of proteins and polysaccharides, imposes a biomechanical resistance that moving cells need to overcome. To invade cells might either degrade the ECM to pass through or modify their shape and squeeze through the ECM pores due to weak adhesion to the ECM and reduced or absent proteolytic activity^44^. These two distinct invasion modes are commonly termed “path-generating” radial (mesenchymal) and “path-finding” amoeboid mode^45^. Both invasion patterns are common for tumor cells to spread and a switch between them is a mechanism for an effective cancer cell invasion^44–49^. The vast majority of Sw71 showed the classical “path-generating” invasion pattern with well-developed invadosomes. Seems that the invasion/migration of Sw71 models is their intrinsic property although there is data showing that the contact with endometrial stromal cells promotes the trophoblast invasive activity^22^⁠. Invading Sw71 spheroids present a useful platform for generation and investigation of invadosomes and their role in implantation. The invadosomes are dynamic protrusions of the plasma membrane found in both normal (podosomes) and in cancer cells (invadopodia)^50–52^. Such formations are complex molecular structures composed of a dense filamentous (F)-actin, actin-regulating proteins, and proteins involved in regulation, adhesion, and scaffolding through their metalloprotease activity^53^⁠. Interestingly, live cell imaging combined with fluorescence microscopy showed that the presence of podosomes correspond with the uptake of antigens and a study revealed podosome formation in response to TGF-β or PMA, two molecules known to stimulate cell migration^54^. When compared to an extended cultured human embryo our 3D Sw71 model for trophoblast migration have shown many similarities like the smeared periphery due to migrating trophoblasts, the establishment of a “primitive syncytium” zone right around the spheroid where single cells could not be easily distinguished, and of a peripheral zone with single differentiated migrating trophoblast cells resembling EVTs^55^. Interestingly, the trophoblast cells migrate well in reduced or even no serum media, showing that the serum level is not the only determinant of migration. Moreover, Sw71 cells are programmed to migrate as they originated from 1st trimester trophoblast (placental) cells and migration is crucial for effective placentation^41,56^⁠. Since serum is rich in many factors for the cell growth and development hsDMEM/F12 represents good positive control to study trophoblast migration. However, here we show that Opti-MEM^™^ could be chosen as a basic condition for further analyses. Unfortunately, the actual serum content in this commercial formulation is unknown^57^ which hinder any conclusions on this factor. Because of the known serum level and composition, the lsDMEM/F12 media may be used for collection of CM for further protein analyses since it would not interfere with the results as much as hsDMEM/F12. The serum-free media is a rather stress inducing condition, far from the physiological state, that may be used for investigation of stress factors secreted from the trophoblasts. The invading trophoblasts (EVT) is known to express a particular combination of three HLA class I molecules—HLA-G, HLA-C and HLA-E^6–8^. The non-classical HLA-G molecule is a unique marker of the trophoblasts and a central component of fetus-induced immune tolerance during pregnancy though interaction with decidual NK cells^6,58^. There are also suggestions for HLA-G providing a tolerogenic signal to the regulatory T cells via presentation to the uterine dendritic cells^59^. The trophoblast HLA-C and HLA-E molecules were shown as dominant ligands for NK cells^7,8^. While the HLA-E is rather monomorphic and would be considered as ‘self’ by maternal immune cells^7^, the polymorphic HLA-C is the only classical HLA class I molecule expressed by the trophoblasts able to present paternal antigens, as well as pathogens at maternal–fetal interface^8,9,60,61^. Via binding to KIRs on the maternal cytotoxic cells this molecule could influence the pregnancy outcome generating the tolerance or immunity ^8,9,58,59^. Thus, the expression of HLA-C by Sw71 spheroid models was of particular interest if we would like to explore them as a model in the immune HLA-C-KIR interactions. The novel finding in this study is the expression/secretion of HLA-C isoforms by the Sw71 spheroids. The similar production of sHLA-C by both Sw71 spheroids and early placenta-derived explants validate the in vivo relevance of the models.

Altogether we demonstrate that Sw71 spheroids as 3D in vitro model is a biologically relevant, useful tool for platforms for evaluation of trophectoderm behavior and functionality during the in vivo concealed process of human implantation. The 3D Sw71 models are easy to handle, select and transfer for functional assays in small volumes, where treated with different factors they can be monitored and analyzed by microscopy and with life cell imaging systems. Of note, any of the other components in the functionality assays may be replaced by primary culture or culture product for further validation. Sw71 spheroids may serve as an effective embryo surrogate for the evaluation of maternal–trophoblast immune interactions through the HLA-C-KIRs axis in (un)successful human implantation. At last, but not least the 3D models are cost effective and relatively easy to be implemented in standard equipped cell culture laboratories.

## Materials and methods

### Cell lines and media

Human first trimester trophoblast cells (Swan 71, Sw.71) and human endometrial epithelial cells (HEC‐1A, ATCC^®^HTB‐112^™^) were used in this study. Sw71 non-tumor cell line for the generation of the 3D models was isolated from the first trimester (7th gestational week (gw)) normal human placenta. Its variant transfected with green fluorescence protein (Sw71-GFP)^20,21^ was used in some of the experiments. For propagation of Sw71 cells and Sw71 spheroid differentiation DMEM‐F12 complete medium i.e., supplemented with 10% heat‐inactivated fetal calf serum (FCS), 10 mmol/L HEPES, 0.1 mmol/L MEM non‐essential amino acids, 1 mmol/L sodium pyruvate, and 100 U/mL penicillin/streptomycin (all from Gibco) was used. HEC‐1A cell line was used to mimic the endometrial epithelium and was grown in McCoy's 5A medium (ATCC), supplemented with 10% FCS, 10 mmol/L HEPES, 0.1 mmol/L MEM non‐essential amino acids, 1 mmol/L sodium pyruvate and 100 U/mL penicillin/streptomycin. To investigate the impact of the serum levels on Sw71 spheroids functionality, media with different levels of serum were tested, as follows: DMEM/F12 complete with 10% FCS, referred as high-level serum medium (hsDMEM/F12), DMEM/F12 complete with 1% FCS, referred as low-level serum medium (lsDMEM/F12), DMEM/F12 complete without serum, referred as serum-free medium (sfDMEM/F12), and commercially available Opti-MEM^™^/GlutaMAX^™^ (Opti-MEM) medium which is with reduced serum content, below 10% (Gibco). All cells were maintained at 37 °C with 95% humidity and 5% CO_2_.

### Differentiation of Sw71 spheroids

For generation of Sw71 spheroids we used scaffold- and agitation-free technique where gravity forced aggregation of Sw71 or Sw71-GFP cells (4 × 10^3^ cells/well) occurs in ultra-low attachment plate (ULA, Costar) after centrifugation (5 min/1500 rpm) and subsequent incubation in DMEM‐F12 complete medium for 48 h. The dynamic process of spheroid differentiation was monitored by time-lapse live cells imaging system OMNI (in brightfield mode) or by Lux3 FL (in both brightfield and fluorescence mode), both systems provided by CytoSmartTM technology (The Netherlands). The detailed brightfield, phase contrast and fluorescent images of the spheroids were taken with ECHO Revolve microscope (RVL-100-M, Echo, San Diego, CA, USA).

### Attachment assay

To mimic the blastocyst-endometrial epithelium contact during attachment we co-cultured differentiated Sw71 spheroids with a confluent monolayer of HEC-1A cells for 24 h. The workflow included: (1) growing of the HEC‐1A cells in monolayer (≥ 95% confluency, 10 000 cells/well for 7 days) in McCoy's 5A complete in 96 well flat-bottomed plate (Costar) and (2) differentiation of Sw71 spheroids in DMEM-F12 complete in ULA plate (Costar) and (3) co-culture for 24 h. The day before spheroid transfer medium was switched to half McCoy’s 5A complete—half medium of interest (hsDMEM/F12, lsDMEM/F12 or Opti-MEM), each condition in triplicate). On the next day differentiated Sw71 spheroids were placed on the top of each HEC-1A monolayered well (4 per well) with wide-bore tips. After imaging the spheroids were left to adhere for 30 min, 1, 2, 4 and 24 h. At each time point the attachment rate (attached vs. placed spheroids) was determined after 3 min (min) shake at 1000 rpm or at 3000 rpm. The force of the shake of 1000 rpm corresponds to ~ 17 Hz frequency, while 3000 rpm—to 50 Hz frequency. Images were taken at each time point with ECHO Revolve microscope (RVL-100-M, Echo, San Diego, CA, USA).

### Invasion and migration assays

Invasion and migration assays were performed using established protocols^22^. Briefly, to measure the three-dimensional invasion of differentiated Sw71 spheroids we transferred them to a flat bottom plate wells covered with solidified 50 μL Matrigel (Sigma Aldrich) in 1:1 ratio with the medium of interest (hsDMEM/F12, lsDMEM/F12 or Opti-MEM), recreating the structure of ECM, covered with 150 μL medium of interest (single spheroid per well, in triplicate for each condition). The invasion was monitored for 6 days, and images were taken every 48 h by ECHO Revolve Microscope (RVL-100-M, Echo, San Diego, CA, USA). The invasion rate (invading vs. tested spheroids) was calculated on D6, and invasion starting at D2 was considered as an optimal. For the migration assay single differentiated Sw71 spheroids were transferred into individual wells of a 96‐well plate containing the medium of interest (hsDMEM/F12, lsDMEM/F12, sfDMEM/F12 or Opti-MEM, 200 μL/well, in triplicates for each condition). The process was monitored for 48 h by live cell imaging systems OMNI or Lux3 FL (CytoSmart^™^ technology, The Netherlands), and was imaged every 24 h with ECHO Revolve microscope (RVL-100-M, Echo, San Diego, CA, USA). The migration rate (migrating vs. tested spheroids) and the migration area were determined on D2. The migration area equals the area without the remaining spheroid surface and the formed lacunae lacking trophoblasts and was measured using ECHO Revolve software.

### Blood samples, placenta samples and trophoblasts explants

Blood samples (n = 12) and early placenta tissue samples (n = 7) were donated by healthy pregnant women in early pregnancy (6–12 gw) directed to elective abortions. Control blood samples (n = 4) were donated by non-pregnant women in reproductive age (volunteers). Gestation tissue specimens were collected in sterile phosphate-buffered saline (PBS) and processed within 1–2 h. Blood samples in serum collection tubes (KABELaboratortechnik) were incubated for 30 min/37 °C for a clot to form and then were centrifuged at 3000 rpm for 10 min, and sera were collected and stored at − 80 °C. We optimized protocol for culture of primary trophoblast derived explants from 1st trimester placenta^62^. Small trophoblast pieces (4–5 mm in diameter) were placed in a 24-well plate or in chamber polystyrene vessel tissue culture treated slide (Falcon) in hsDMEM/F12 complete and cultured for 72 h with daily monitoring for vitality. Conditioned media was collected at 72nd hour, centrifuged to remove any cellular material and stored at − 80 °C until use. All human samples used in this study were obtained in accordance with the guidelines of The Declaration of Helsinki and approved by Human Research Ethics Committee at the University Obstetrics and Gynecology Hospital “Maichin Dom'', Medical University, Sofia, Bulgaria (No. 250569/2018). Written informed consent was taken from all subjects for the use of tissue samples.

### Immunocytochemistry

3D Sw71 models or 2D Sw71 monolayers were cultured for 24–48 h in sterile culture chambers on a glass slide (Falcon). Trophoblast derived explants were cultured in the same type of chambers for expansion of EVTs, and after that the explants were removed carefully. The cells were fixed in 2% paraformaldehyde (PFA) in PBS, overnight (ON) at RT and stained for HLA-C using indirect immunofluorescent method. After washing with PBS, the cells were incubated with 10% goat serum (to block the non-specific binding) and then subsequently with purified polyclonal rabbit anti-human HLA-C antibody (E-AB-17922, Elabscience) and AF488-conjugated goat anti-rabbit antibody (E-AB-1055, Elabscience). Negative controls were prepared by omitting primary antibody and/or secondary antibody. Control staining was done with mouse polyclonal anti-human HLA-G antibody (E-AB-18031, Elabscience) and goat anti-mouse IgG (E-AB-1015, Elabscience). The slides were imaged with ECHO Revolve microscope (RVL-100-M, Echo, San Diego, CA, USA).

### Histology and immunohistochemistry (IHC)

Early pregnancy trophoblast tissues (10 mm × 10 mm × 10 mm) were fixed in formalin‐free HOPE fixative (Innovative Diagnostik‐System, Hamburg, Germany). The samples were processed and embedded in paraffin wax acc. to the manufacture’s recommendations. The paraffin sections (5 μm) were routinely stained with hematoxylin/eosin for histological investigation and then selected slides were subjected to IHC for HLA-C staining using three‐step biotin–streptavidin enzyme method. We used purified rabbit anti‐human HLA-C mAb (E-AB-17922, Elabscience) and UltraTek Anti‐Polyvalent visualization system following the recommendations of the manufacturer (SkyTec Laboratories, Logan, UT, USA). For negative control, the primary antibody was omitted. Sections from human breast cancer tissue processed in the same way were used as positive controls for specificity of HLA-C staining.

### HLA-C ELISA

Conditioned media (CM) was collected from 2 and 3D Sw71 cultures, and lsDMEM/F12 condition was chosen to minimize the serum impact on further HLA-C analyses. 2D Sw71 cultures from different passages, grown ON in hsDMEM/F12 (0.175 * 10^6^ cells per 35 mm petri dish (Corning) were cultured for another 48 h in lsDMEM/F12. Differentiated 3D Sw71 spheroids were transferred to lsDMEM/F12 for another 48 h. As a tissue primary culture that must remain vital for 72 h, the trophoblast explants were cultured in hsDMEM/F12. CM was collected at the corresponding time, centrifuged (3 min/1500 rpm) to remove any cellular debris and stored at − 80 °C until use. The samples were thawed at once and subjected to ELISA for detection of soluble HLA-C using commercial quantitative HLA-C ELISA kit (E-EL-H2331, Elabscience). CM samples were run in duplicate alongside serial dilutions of human HLA-C standard as per the manufacturer’s instructions. Serum samples (diluted 1:200) from pregnant and non-pregnant women served as a positive control for HLA-C detection. The absorption was measured at 450 nm (ELISA Microplate reader LKB 5060-006). Concentration of HLA-C was calculated from the formula gained from the automatic curve fitting using software at https://mycurvefit.com/.

### Statistical analyses

Statistics was processed with GraphPad Prism v.5.0 software using Mann Whitney test (differentiation and ELISA) and One-way ANOVA (migration). Data represent the average of at least three biological replicates and are presented as mean ± SD. Statistical significance is defined as *p* < 0.05 (*); *p* < 0.005 (**); *p* < 0.001 (***).

## Data Availability

The datasets generated during and/or analyzed during the current study are available from the corresponding author on reasonable request.
